# Ascorbate Biosynthesis during Early Fruit Development Is the Main Reason for Its Accumulation in Kiwi

**DOI:** 10.1371/journal.pone.0014281

**Published:** 2010-12-09

**Authors:** Mingjun Li, Fengwang Ma, Dong Liang, Juan Li, Yanlei Wang

**Affiliations:** College of Horticulture, Northwest A&F University, Yangling, China; University College London, United Kingdom

## Abstract

**Background:**

Ascorbic acid (AsA) is a unique antioxidant as well as an enzyme cofactor. Although it has multiple roles in plants, it is unclear how its accumulation is controlled at the expression level, especially in sink tissues. Kiwifruit (*Actinidia*) is well-known for its high ascorbate content. Our objective was to determine whether AsA accumulates in the fruits primarily through biosynthesis or because it is imported from the foliage.

**Methodology/Principal Findings:**

We systematically investigated AsA levels, biosynthetic capacity, and mRNA expression of genes involved in AsA biosynthesis in kiwi (*A*. *deliciosa* cv. Qinmei). Recycling and AsA localization were also monitored during fruit development and among different tissue types. Over time, the amount of AsA, with its capacity for higher biosynthesis and lower recycling, peaked at 30 days after anthesis (DAA), and then decreased markedly up to 60 DAA before declining more slowly. Expression of key genes showed similar patterns of change, except for L-galactono-1,4-lactone dehydrogenase and L-galactose-1-phosphate phosphatase (GPP). However, GPP had good correlation with the rate of AsA accumulation. The expression of these genes could be detected in phloem of stem as well as petiole of leaf and fruit. Additionally, fruit petioles had greater ascorbate amounts, although that was the site of lowest expression by most genes. Fruit microtubule tissues also had higher AsA. However, exogenous applications of AsA to those petioles did not lead to its transport into fruits, and distribution of ascorbate was cell-specific in the fruits, with more accumulation occurring in larger cells.

**Conclusions:**

These results suggest that AsA biosynthesis in kiwi during early fruit development is the main reason for its accumulation in the fruits. We also postulate here that GPP is a good candidate for regulating AsA biosynthesis whereas GDP-L-galactose-1-phosphate phosphorylase is not.

## Introduction

**Figure 1:**
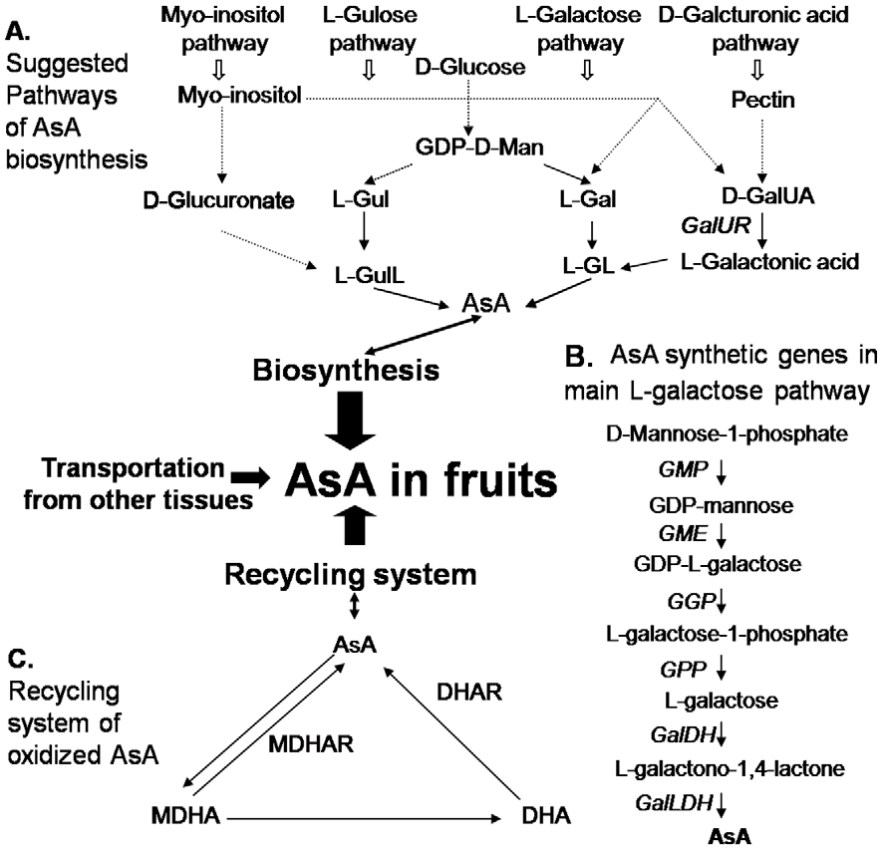
Possible schemes for ascorbate accumulation in fruits [7],[23]. (A) suggested pathways of AsA biosynthesis. D-Glu, D-glucose; L-Gal, L-galactose; L-GL, L-galactono-1,4-lactone; L-Gul, L-gulose; L-GulL, L-gulono-1,4-lactone; D-GalUR, D-galacturonate reductase, GDP-D-Man, GDP-D-mannose. (B) AsA synthesis genes in main L-galactose pathway. GMP, GDP-mannose pyrophosphorylase; GME, GDP-mannose-3′,5′-epimerase; GGP, GDP-L-galactose-1-phosphate phosphorylase; GPP, L-galactose-1-phosphate phosphatase; GalDH, L-galactose dehydrogenase; GalLDH, L-galactono-1,4-lactone dehydrogenase. (C) recycling system for oxidized AsA. MDHA, monodehydroascorbate; MDHAR, monodehydroascorbate reductase; DHA, dehydroascorbate; DHAR, dehydroascorbate reductase.

L-Ascorbic acid (AsA), also called Vitamin C or ascorbate, is one of the most abundant antioxidants, and is a cofactor for many dioxygenases in plants. Because of these unique functions as well as its benefits to human health, increasing attention has been paid to AsA synthesis and regulation in plant tissues [1]–[3]. *De novo* biosynthesis is believed to be a main reason for its accumulation in plant cells. A pathway for AsA formation has also been completely characterized in animal systems [4]. This pathway involves D-glucose as the initial precursor, with the last step being catalysis by L-gulono-1, 4-lactone oxidase, which oxidizes L-gulono-1, 4-lactone (L-GulL) to produce AsA. Only recently has strong evidence been reported for a novel pathway in plants that differs from that in animals [5]. Phenotypic analysis of mutant or transgenic plants has demonstrated the operation of the L-galactose or Smirnoff–Wheeler pathway as a main AsA biosynthetic route [5]–[9]. There, ascorbate can be synthesized from D-mannose-1-phosphate via GDP-mannose and GDP-L-galactose (GDP-L-Gal). Free L-galactose (L-Gal) is released from GDP-L-Gal through the action of GDP-L-Gal phosphorylase (GGP) [10] and L-galactose -1-phosphate phosphatase (GPP) [6]. It is then oxidized by L-Gal dehydrogenase (GalDH) to form L-galactono-1,4-lactone (L-GL) [5]. L-GL is oxidized to AsA by L-galactono-1, 4-lactone dehydrogenase (GalLDH) (Figure 1A, C).

Based on biochemical and genetics studies, three alternative AsA biosynthetic pathways have been proposed (Figure 1A). These include the D-galacturonic acid pathway, which utilizes D-galacturonic acid for the synthesis of L-galacturonic acid derivatives via D-galacturonate reductase (GalUR). This also requires GalLDH to produce AsA in the last step [11]. Second, the L-gulose pathway utilizes L-gulonic derivatives and is a branch of the L-galactose pathway [12]. Finally, the myo-inositol pathway synthesizes L-gulonic derivatives from myo-inositol (MI) [13], [14]. All of these pathways possibly cooperate with the L-galactose pathway or else work in different tissues or under various physiological conditions [15]. AsA translocation from source to sink tissues has been demonstrated in a range of plants, including *Arabidopsis thaliana*, *Medicago sativa* [16], and *Solanum tuberosum* [17]. Its biosynthesis also has been reported to occur within phloem strands [18].

When formed, AsA is not a stable metabolic product but can be oxidized to monodehydroascorbate radicals (MDHA) and dehydroascorbate (DHA), functioning as a major antioxidant to scavenge reactive oxygen species or as an enzymatic cofactor [19]. The resulting MDHA and DHA can be enzymatically reduced to AsA by NADPH- or NADH-dependent monodehydroascorbate reductase (MDHAR; EC 1.6.5.4) and dehydroascorbate reductase (DHAR; EC 1.8.5.1) respectively (Figure 1C) [19]. The importance of MDHAR and DHAR in regulating AsA levels has been shown in transgenic plants by expressing enzymes involved in the recycling of oxidized AsA, including DHAR [20] and MDHAR [21].

Fruits and vegetables are the major sources of AsA for the human system, which is unable to synthesize it but must secure it through dietary uptake [22]. Consequently, researchers need an improved understanding of how ascorbate levels are controlled in fruits. Although the genetics and biochemistry of AsA biosynthesis and information about its physiological roles have been reported for photosynthetic tissues [15], [23], few studies have focused on the mechanism for its molecular regulation and accumulation in sink tissues (e.g., tubers and fruits).

Kiwifruit (*Actinidia*), one of the most popular fruits, is highly regarded for its high AsA content. Ascorbate synthesis is known to occur within the fruits themselves [2], such as acerola [24], blackcurrant [25], and apple [26]. However, AsA biosynthesis has not been characterized in kiwi fruit. Although its translocation has been demonstrated from source to sink tissues [16], [17], researchers have not previously determined whether AsA accumulation in the fruits results mainly from synthesis *in situ* or via import from the foliage. Bulley *et al.* [2] have suggested that the transcript level of *GGP* in various genotypes of kiwi is a major control point because its expression is correlated with AsA content during fruit development. Furthermore, overexpression of that gene leads to an increased AsA supply in *Arabidopsis* plants. However, GGP transcripts are not correlated with AsA content and accumulation rate whereas those of GPP are strongly correlated in tomato fruits [3] and apple leaves [27]. Therefore, the relationship between GGP or GPP to AsA in kiwi fruits requires further analysis.

To gain new insights into the regulatory mechanisms for AsA accumulation in the fruits of kiwi, we performed a systematic investigation of AsA levels, mRNA expression of genes involved in its biosynthesis, and the activities of enzymes and recycling processes over time and among different tissues (especially conducting tissues). We also examined the relationship between ascorbate in fruit petioles and flesh and its distribution in the fruits. Our objectives were to 1) obtain information useful to breeding programs that focus on improving AsA contents in fruits, and 2) elucidate the mechanisms that regulate AsA accumulations in the cells of sink organs.

## Materials and Methods

### Plant materials

Six-year-old vines of kiwi (*Actinidia deliciosa* cv. Qinmei) were trained as a trellis system and grown at a 2×3 m spacing in an experimental orchard at the Horticultural Experimental Station of Northwest A & F University, Yangling, China (34°20′N, 108°24′E). Fruits were harvested at 15-d intervals following anthesis (±3 d; DAA, days after anthesis), between 4 and 5 PM. The day on which the petals had just dropped off was designated as 0 DAA. Mature leaves also were collected at each time point. Fruits were directly sampled through their transverse centers, using a cork borer (0.5-cm diam.). They were immediately frozen in liquid nitrogen and stored at −80°C. At each collection time point, four replications were made, with each comprising six fruits from the same vine.

To evaluate the relationship between AsA levels and expression of related genes in conducting tissues within the same tree at 40 DAA, we obtained mature leaves, young leaves (leaf area  = 1.0 to 1.5 cm^2^), petioles from leaves and fruits, and phloem from branch samples (four replications). All were immediately frozen in liquid nitrogen and stored at −80°C.

To determine whether AsA contents are correlated between fruit petioles and flesh, we performed two independent experiments. First, fruits with their petioles were harvested at about 28 DAA and 140 DAA, and placed on ice. In the lab, the petiole nodes were cut under water, then inserted into a 10 mM AsA solution and cultured for 24 h in a pre-humidified atmosphere at 20°C. Samples of flesh and core were also measured for AsA levels in this manner while control samples were cultured only in water. The second experiment, done with *in vivo* tissues, entailed lightly scratching the surface of the fruit petioles with a hacksaw blade at about 15 DAA and 130 DAA. The petioles were immediately washed with water, then wrapped with cotton cord. Afterward, 5 mM AsA, DHA, or L-GL (each dissolved in 20 mM MES buffer, pH 6.0) was applied to the cord before covering the tissue with film to preserve freshness. At 3-d intervals, 5 mM AsA, DHA, or L-GL was again added; fruits and petioles were harvested after 15 d of this treatment. As our control, 20 mM MES buffer (pH 6.0) was applied alone.

### Assays for AsA

Samples of fruit (2 g each) or leaves (0.5 g) were homogenized in 8 mL of ice-cold 6% (v/v) HClO_4_ and centrifuged at 12,000 *g* for 20 min at 2°C. Hepes buffer (0.1 M; pH 7.0) was added to the extracts at a 1∶5 ratio (buffer:extract, v:v); K_2_CO_3_ (5 M) was then added in until the pH reached 5.6. Extracts were centrifuged again at 12,000 *g* for 2 min to allow the removal of precipitated K_2_ClO_4_, and the supernatants were used to assay total ascorbate (AsA + DHA, T-AsA), AsA and DHA as described by Ma and Cheng [28]. That assay is based on the oxidation of AsA by ascorbate oxidase in an acidic solution. AsA was calculated as the difference in absorption at 265 nm before and after the addition of ascorbate oxidase. Its concentration was quantified by comparing the difference in absorption relative to a standard curve.

### AsA biosynthesis tested by feeding with candidate precursors

Fruits were sampled at 30, 75, and 125 DAA by removing fresh tissues with a cork borer (1-cm diam.) and cutting them into 0.3-cm-thick discs. Simultaneously, 1-cm-diameter leaf discs were prepared from mature leaves. As described by Hancock *et al.* [18], all samples were pre-incubated at 25°C for 1 h in 25 ml of buffer containing 20 mM MES (pH 5.5), 300 mM mannitol, 5 mM MgCl_2_, 2 mM KCl, 1 mM CaCl_2_, and 1 mM CaSO_4_, on a rotary shaker at 100 rpm. Afterward, precursors D-glucose (D-Glu), L-galactono-1,4-lactone (L-GL), L-galactose (L-Gal), L-gulono-1,4-lactone (L-GulL), myo-inositol (MI), D-glucuronic acid (D-GluA), or D-galacturonic acid (D-GalUA) were added, to a final concentration of 10 mM for each treatment solution. As our control, sucrose was used instead of a precursor. Following incubation for 20 h on a rotary shaker (100 rpm) at 25°C, these samples were washed with sterile water and surface-dried on filter paper, then immersed in liquid nitrogen. Their AsA concentrations were determined as described above.

### mRNA expression analysis

Expression of the genes involved in AsA biosynthesis and recycling was evaluated by quantitative reverse transcription-polymerase chain reactions (qRT-PCR). We cloned the full-length open reading frames of *GalDH* (GenBank EU525846), *GGP* (GenBank GU339036), *GME* (GenBank GU339037), *GMP* (GenBank FJ643600), *GalUR1* (GenBank GU339035), and *DHAR1* (GenBank GU339034), as well as the partial sequences of *GalLDH* (GenBank GU339039), *GalUR2* (GenBank GU339038), *DHAR2* (GenBank GU339040), and *MDHAR* (GenBank GU339041) via RT-PCR. *GPP* (GenBank AY787585) had been previously described by Laing et al. [29]. The partial sequence of *GalUR3* (GenBank FG4889261) found in *Actinidia* ESTs showed high homology with strawberry GalUR (GenBank AF039182) [11]. Gene-specific primers (Table S1) were designed from these sequences of kiwifruit genes, using Primer5 software. Total RNA was extracted from samples by the modified CTAB method [30], and DNase was used to clean out DNA before reverse-transcription began. qRT-PCR was performed with a PrimeScriptTMRT Reagent Kit (Takara) plus oligo(dT)20 and random primers for cDNA synthesis, according to the manufacturer's protocol. The amplified PCR products were quantified by an iQ5 Multicolor Real-Time PCR Detection System (Bio-Rad Laboratories, Hercules, CA, USA), with a SYBR Premix Ex Taq kit (Takara). *Actin1* (GenBank EF063572) transcripts were utilized to standardize the different gene cDNA samples throughout the text. *Actin1* conservation was detected by utilizing *18S ribosomal RNA* (GenBank AB253775) as our control. For all samples, four tubes of total RNA were extracted from each of four replications, and then mixed in another tube for reverse-transcription. qRT-PCR experiments were done with four technical replications. All data were analyzed by the ddCT method, with iQ5 2.0 standard optical system analysis software.

Semi-quantitative RT-PCR was conducted to determine expression; cDNA was subject to PCR with primers specific for each candidate gene. For almost all PCR reactions, 30 cycles were used; the exception was 33 cycles for *GME* and *GalLDH* because of their relatively low expression. The *actin1* gene served as our internal control, for which 32 cycles were run. In subsequent PCR reactions, one RNA sample without an RT reaction was used as a negative control.

### Assays of GalLDH and GalDH activities

Crude GalLDH (EC 1.3.2.3) enzyme extract was prepared according to the method of Ôba et al. [31], with some modifications. For GalLDH, samples of fruits (5 g) or leaves (2 g) were homogenized in 30 ml of 50 mM potassium phosphate buffer (pH 7.5) containing 0.4 M sucrose, 10% (v/v) glycerol, 0.1 mM phenylmethanesulfonyl fluoride, 0.2% (v/v) Triton X-100, and 0.3% (v/v) mercaptoethanol; 1 g of cross-linking polyvinylpyrrolidone (PVP) was added because kiwi fruit has a higher sugar content. The homogenate was centrifuged at 500 *g* for 10 min at 2°C, and the supernatant was centrifuged at 16,000 *g* for 40 min at 2°C. The pellet was suspended in 2 ml of 0.1 M phosphate buffer (pH 8.0) that contained 5 mM glutathione, 1 mM EDTA, and 10% (v/v) glycerol. This suspended solution was again centrifuged at 2,000 *g* for 10 min at 2°C, and the supernatant was utilized to determine the activity of GalLDH after cytochrome c was reduced. A reaction mixture (1.5 ml), containing 60 µM cytochrome c, 1 mM sodium azide, 2.5 mM L-GL, 0.1% (v/v) Triton X-100, and 0.1 mL of the enzyme extract in 50 mM Tris-HCl (pH 8.5), was pre-incubated at 27°C for 5 min. Subsequently, reduction of cytochrome c was monitored by the increase in absorption at 550 nm. One unit of activity was defined as the reduction of 1 µmol of cytochrome c per minute.

GalDH (EC 1.1.1.117) activity was assayed according to a method described by Gatzek et al. [8], with some modifications. Samples were extracted on ice in 0.1 M potassium phosphate buffer (pH 7.0) containing 0.1 mM phenylmethanesulfonyl fluoride, 0.5% (v/v) Triton X-100, 0.2% (v/v) 2-mercaptoethanol, and 2% (w/v) PVP-4000. The homogenates were centrifuged at 16,000 *g* for 20 min at 2°C and the supernatants were collected as enzyme extracts. A reaction mixture (1.5 ml), containing 0.5 mM NAD^+^, 1 mM L-Gal, and 0.1 mL of the supernatant in 100 mM Tris-HCl (pH 8.0), was pre-incubated at 27°C for 2 min. Afterward, reduction of NAD^+^ was monitored by the increase in absorption at 340 nm. Activity was calculated in terms of µmol of NAD^+^ reduced per minute.

### Assays of DHAR and MDHAR activities

DHAR (EC 1.8.5.1) and MDHAR (EC 1.6.5.4) were assayed using the method described by Ma and Cheng [28]. Samples (2 g) were homogenized with 8 mL of 50 mM potassium phosphate buffer (pH 7.5) containing 1 mM EDTA, 1 mM DTT, 0.1% (v/v) Triton X-100, and 2% (w/v) mercaptoethanol, with 0.5 g cross-linking PVP. The homogenates were centrifuged at 16,000 *g* for 20 min at 4°C and the supernatants were collected for enzyme assays. Proteins were measured according to the method of Bradford [32], using bovine serum albumin as a standard. MDHAR activity was assayed at 340 nm in 3 ml reaction mixture containing 50 mM Hepes–KOH (pH 7.6), 0.1 mM NADH, 0.25 mM AsA, 0.25 units AsA oxidase, and 0.1 ml of the supernatants. The reaction was initiated by adding AsA oxidase. DHAR activity was measured at 265 nm in 3 ml of assay solution containing 100 mM Hepes–KOH (pH 7.0), 1 mM EDTA, 2.5 mM GSH, 0.2 mM DHA, and 0.1 ml of the supernatants. The reaction was initiated by adding DHA. MDHAR activity was calculated in terms of µmol of NADH oxidized per minute, while that of DHAR was expressed as µmol of AsA produced per minute.

### Detection of AsA in the phloem exudates

Phloem exudates were collected from the petioles of source leaves or 30-DAA fruits, using an adaptation of the method developed by King and Zeevart [33]. Following excision, a 5-mm portion of the petiole was removed under water; then a 20-mm-long segment was taken and rinsed and its cut end was transferred to a 2.5-ml reaction tube containing 500 µl of 10 mM EDTA (pH 7.5). The petiole samples were placed in a refrigerator at 4°C and their exudates were collected for 24 h in the dark. Control samples, run in parallel, entailed petioles that were incubated in 5 mM CaCl_2_ (pH 7.5) to induce callose gellation and to reduce exudation [33]. Following incubation, 500 µl of 10% (w/v) trichloroacetic acid was added before the samples were centrifuged (16000 g, 1°C, 5 min). AsA content was determined as described above.

### AsA localization

Ascorbate was localized in 55-DAA fruits as described by Chinoy [34]. Briefly, transverse and longitudinal sections (approx. 2.5 mm thick) were removed with a blade, then washed with distilled water. Sections were incubated in a 5% AgNO_3_ solution containing a mixture of distilled water:methanol:acetic acid (29∶66∶10, v∶v∶v). After incubation at 4°C for 20 h, that solution was replaced with a 70% methanol solution containing 5% ammonium to stop the reaction. This color reaction was recorded with a Canon S80 camera.

To assess AsA distribution at the cellular level, samples from the flesh, core, and microtubule zone were taken from fruit for which AsA had been localized by the cool acidic-alcoholic AgNO_3_ method (above). They were fixed in FAA solution, dehydrated in a t-butyl alcohol series, then embedded in paraffin. Sections (10 µm) were stained with safranin and fast green and photographed under a light microscope (Dialux 22; Leitz, Wetzlar, Germany).

### Statistical analysis

Each sample was replicated at least four times, with each replication measured twice. Results were represented as means ± standard deviation (SD). Significant differences were calculated by a Duncan's means test at the *P*<0.05 level.

## Results

### Changes in AsA levels during kiwi fruit development

**Figure 2:**
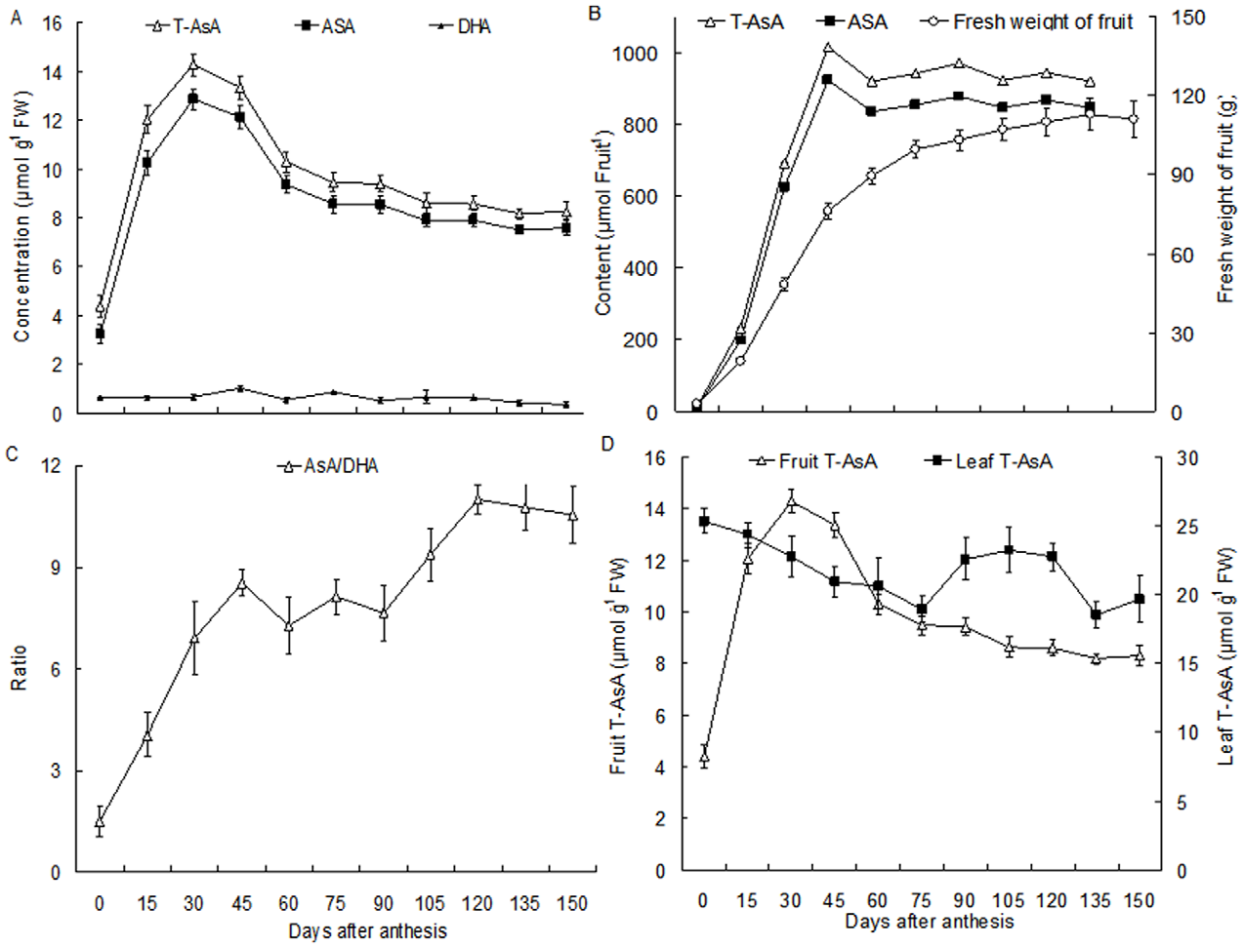
Changes in AsA levels and accumulation during kiwi fruit development. (A) T-AsA, AsA, and DHA concentrations per fresh weight; (B) AsA/DHA ratio; (C) T-AsA and AsA accumulations per fruit; (D) relationship of T-AsA concentrations between fruits and leaves. Values are means of at least 4 replicates ± SD.

AsA levels were monitored from young *Actinidia* fruit up to the stage of physiological ripening. Based on fresh weights, contents of T-AsA and AsA were lowest in 0-DAA fruits, and then increased rapidly to a peak at 30 DAA before decreasing. Levels clearly declined in 60-DAA fruits versus those at 45 DAA before decreasing slightly as the fruit matured (Figure 2A). In contrast, AsA/DHA values increased markedly from 0 to 45 DAA, where they were maintained before rising again from 90 to 120 DAA (Figure 2B). T-AsA and AsA accumulations were highest at 45 DAA, and were not further enhanced as fruit mass increased (Figure 2C). By comparison, changes in leaf AsA levels did not follow the trend seen for fruits harvested at those same time points (Figure 2D).

### Feeding with precursor candidates for AsA biosynthesis in developing fruits

**Figure 3:**
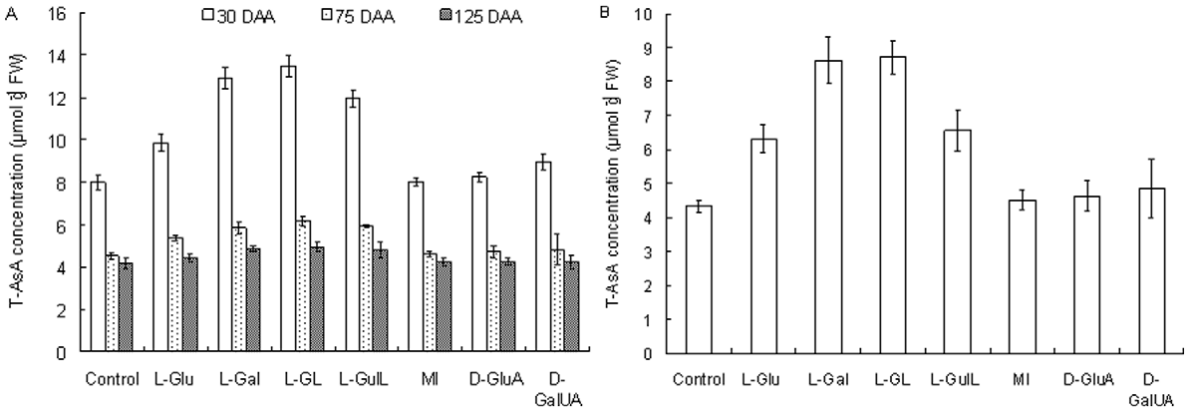
Effects of possible precursors for AsA biosynthesis on AsA content in mature leaves and flesh of fruits at different developmental stages. Control was sucrose rather than precursor. (A) T-AsA concentration in fruits over time. (B) T-AsA concentration in treated leaves. Values are means of 4 replicates ± SD.

To understand the fluctuation in AsA biosynthesis at different developmental stages, we treated flesh discs *in vitro* with precursor candidates. For 30-DAA fruit, feeding with D-Glu, L-GL, L-Gal, or L-GulL elevated T-AsA contents by approximately 1.2-, 1.6-, 1.7-, or 1.5-fold, respectively, compared with the control; only slight increases in contents were observed with 75- and 125-DAA fruits (Figure 3A, B). By contrast, no clear effect was found with MI and D-GluA, whereas feeding with D-GalUA led to only a slight rise in T-AsA content in 30-DAA fruits. The same overall pattern from 30-DAA fruit was seen for leaves, with L-GL, L-Gal, and L-GulL contributing to an obvious improvement in T-AsA contents (Figure 3C, D).

### Changes in mRNA expression of genes involved in AsA synthesis and recycling during fruit development

**Figure 4:**
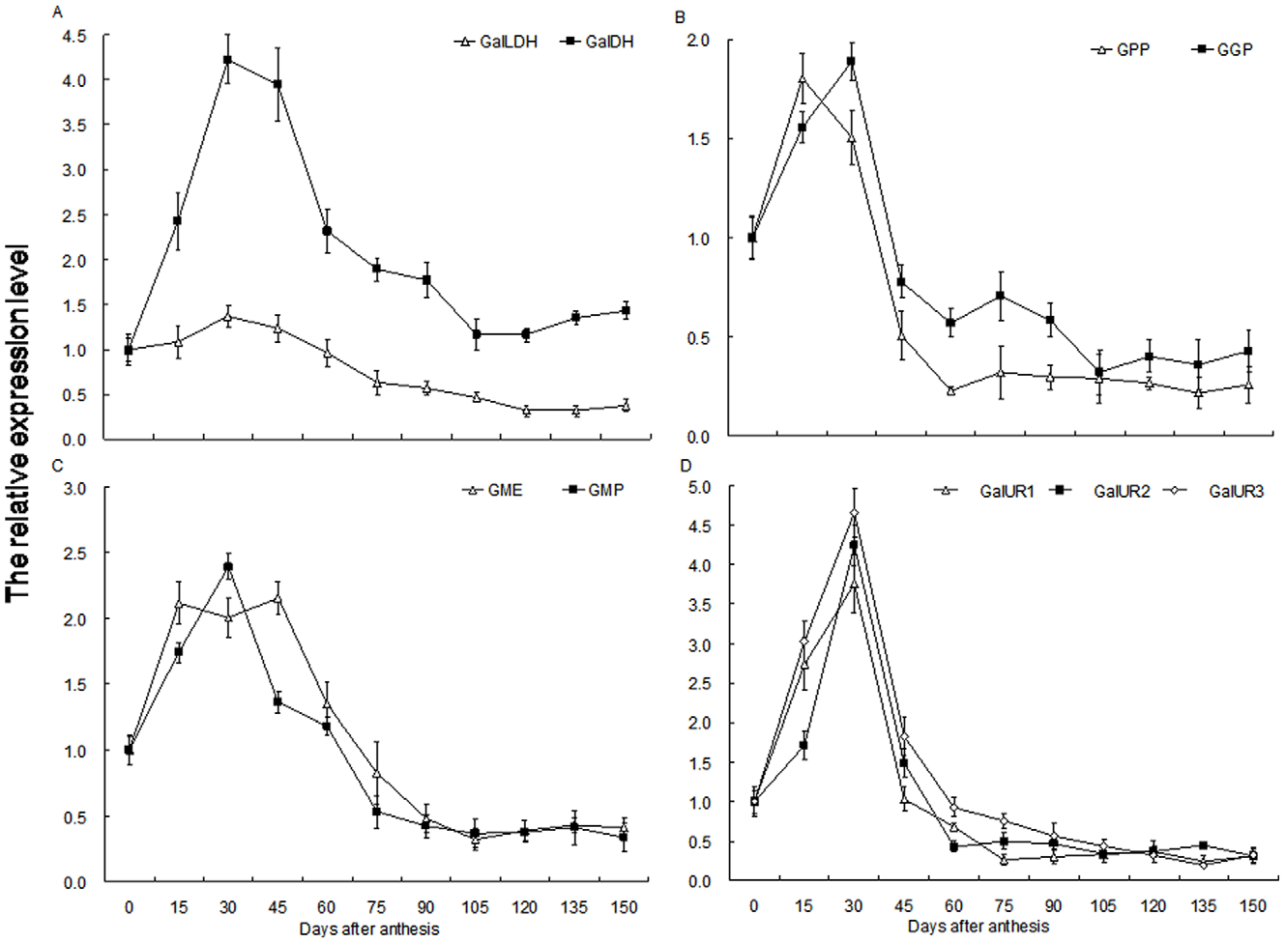
Changes in mRNA relative expression of genes involved in AsA biosynthesis during fruit development. (A) *GalLDH* and *GalDH*; (B) *GPP* and *GGP*; (C) *GME* and *GMP*; (D) *GalUR1*, *GalUR2*, and *GalUR3*. Total RNA was extracted from fruits and quantitative RT-PCR was performed with specific primers designed from coding sequences of *GalLDH*, *GalDH*, *GPP*, *GGP*, *GME*, *GMP*, and *GalUR*. For each sample, transcript levels were normalized with those of *Actin1* (control); expression over time was determined relative to a designation of ‘1’ at 0 DAA. Values are means of at least 3 replicates ± SD.

RT-qPCR was used to investigate the degree of mRNA expression by *GalLDH*, *GalDH*, *GPP*, *GGP*, *GME*, and *GMP*, all of which are involved in the L-galactose pathway for AsA biosynthesis. Three *GalURs* also provided the main evidence for a complete D-galacturonate pathway during fruit development. Here, *actin1* served as our internal standard (Figure 4). Relative expression levels of *GalLDH* showed little increase before 30 DAA, at which time they then peaked (Figure 4A) before decreasing gradually until 120 DAA (Figure 4A). Expression of *GalDH* was most abundant in 30-DAA fruits, with transcripts being maintained at that point to 45 DAA. Afterward, levels decreased through 60 DAA after which they remained largely unchanged from 105 DAA onward (Figure 4A).

The relative expression of *GPP* peaked in 15-DAA fruits, and then clearly declined to a steady level after 60 DAA; from Day 30 to 45, expression decreased by about 65% (Figure 4B). This was similar to the pattern of change we had noted for the rate of AsA accumulation during fruit development. *GGP* expression was highest at 30 DAA, then decreased greatly at 45 DAA before remaining largely unchanged through fruit maturation (Figure 4B). Transcript levels for *GME* were highest from 15 to 45 DAA before decreasing to 105 DAA (Figure 4C). After peaking in 30-DAA fruits, the relative expression of *GMP* dropped toward Day 75 and then remained fairly constant toward maturation (Figure 4C). Relative mRNA levels were similar over time among *GalUR1*, *GalUR2*, and *GalUR3*. Their transcripts rapidly reached their peak at 30 DAA, followed by a clear decline at 45 DAA before remaining mostly steady after Day 60 (Figure 4D).

**Figure 5:**
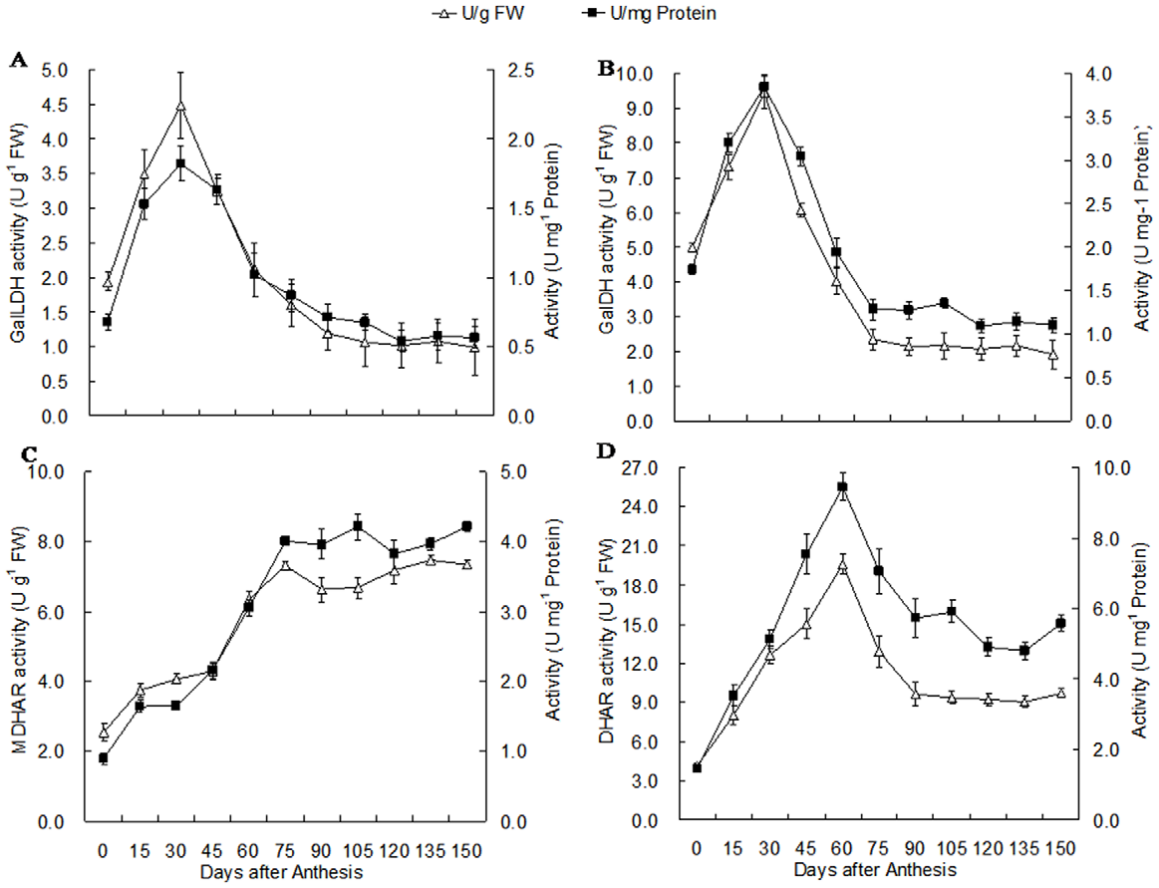
Changes in mRNA relative expression of genes involved in AsA recycling during fruit development. (A) *MDHAR*; (B) *DHAR1* and *DHAR2*. Total RNA was extracted from fruits and quantitative RT-PCR was performed with specific primers designed from coding sequences of *MDHAR*, *DHAR1*, and *DHAR2*. For each sample, transcript levels were normalized with those of *Actin1* (control); expression over time was determined relative to a designation of ‘1’ at 0 DAA. Values are means of at least 3 replicates ± SD.

*MDHAR* and *DHAR* encode two key enzymes for recycling AsA. Here, relative expression of *MDHAR* mRNA showed no obvious changes from 0 to 60 DAA, but was clearly increased toward 90 DAA, after which it remained at a constant level throughout maturation (Figure 5A). Transcripts of *DHAR1* increased rapidly for the first 30 d, and were maintained up to 75 DAA. This was followed by a dramatic drop at 90 DAA, but then no further changes. *DHAR2* was markedly increased to its highest abundance at 45 DAA, and declined gradually to its original level at 105 DAA, where it remained largely unchanged while the fruit matured (Figure 5B).

### Changes in activities of key enzymes involved in AsA synthesis and recycling during fruit development

**Figure 6:**
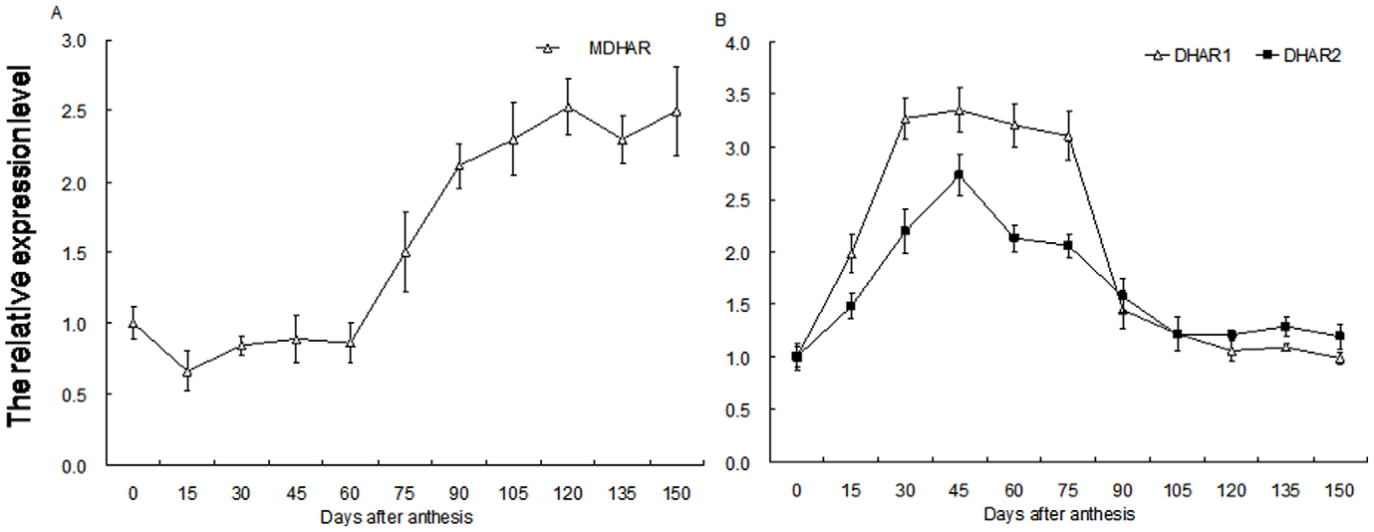
Changes in activities of GalLDH (A), GalDH (B), MDHAR (C) and DHAR (D) during fruit development. Values are means of at least 4 replicates ± SD.

On a fresh-weight basis, the patterns of GalLDH activity did not follow those manifested by its relative mRNA expression (Figures 4A, 6A). Peaking at 30 DAA, such activity showed distinct decreases at 45 and 90 DAA, before slowing over time. Based on protein content, however, GalLDH activity had a clearer peak at 30 DAA compared with values calculated per fresh weight (Figure 6A). For GalDH, the greatest peak, based on fresh weight, was reached at 30 DAA, followed by a rapid decrease to a steady level after 75 DAA (Figure 6B). GalDH activity, when based on protein content as well, also was highest at 30 DAA (Figure 6B).

On a fresh-weight basis, MDHAR activities markedly increased to their highest level at 60 DAA, where they remained constant toward maturation. Based on protein content, however, such activity did not peak until 75 DAA (Figure 6C). For DHAR, activity was greatest at 60 DAA, then clearly decreased toward 90 DAA and remained unchanged to 150 DAA (Figure 6B).

### Differences in AsA levels and relative mRNA expression in various tissue types

We compared AsA levels among sinks (young fruits and leaves), sources (mature leaves), and conducting tissues (leaf and fruit petioles, and phloem). T-AsA and AsA contents were highest in mature leaves, with T-AsA being about 1.55- and 1.80-fold greater than in young fruits and leaves, respectively (Table 1). In the conducting tissues, fruit petioles had the most T-AsA, about 49% of that measured in young fruits. In leaf petioles and the phloem, those contents were about 15% and 24%, respectively, of that within young fruits. Those immature fruits also had the highest ratio of AsA/DHA, followed by the leaves; no clear difference was found among conducting tissues.

**Table 1:** Comparison of AsA levels among different tissues of kiwifruit sampled at 30 DAA.

	T-AsAconcentration(µmol·g−1·FW)	AsAconcentration(µmol·g−1·FW)	DHAconcentration(µmol·g−1·FW)	AsA/DHA ratio
Young fruit	12.12±0.65b	10.85±0.48b	1.27±0.14d	8.54±0.36a
Mature leaf	18.75±0.92a	14.12±0.58a	4.63±0.35a	3.05±0.22b
Young leaf	10.25±0.42c	7.84±0.37c	2.41±0.17b	3.25±0.16b
Leaf petiole	1.87±0.18f	1.35±0.13f	0.52±0.18e	2.59±0.24c
Fruit petiole	6.88±0.36d	5.01±0.18d	1.87±0.19c	2.67±0.14c
Phloem	3.23±0.13e	2.29±0.19e	0.94±0.18d	2.44±0.22c

Values of AsA levels are means of 5 replicates ± SD. Different letters within the same column indicate significant difference at P<0.05 by Duncan's test.

**Figure 7:**
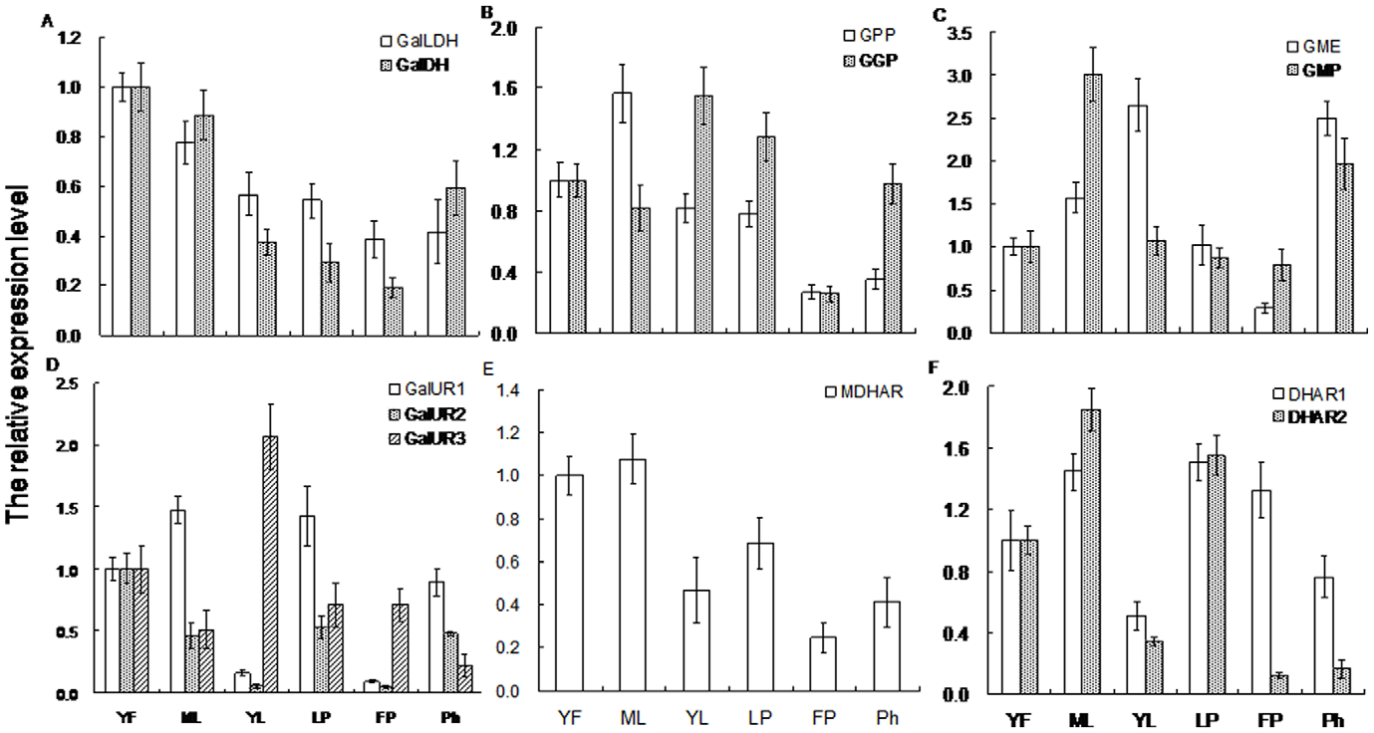
Differences in mRNA relative expression of genes involved in AsA biosynthesis and recycling among tissue types. (A) *GalLDH* and *GalDH*; (B) *GPP* and *GGP*; (C) *GME* and *GMP*; (D) *GalUR1*, *GalUR2*, and *GalUR3*; **E**, *MDHAR*; **F**, *DHAR1* and *DHAR2*. YF, young fruit (30 DAA); ML, mature leaf; YL, young leaf; LP, leaf petiole; FP, fruit petiole; Ph, phloem. Total RNA was extracted from samples harvested at 30 DAA. Quantitative RT-PCR was performed with specific primers designed from coding sequences of kiwifruit. For each sample, transcript levels were normalized with those of *Actin1*, and expression across tissue types was determined relative to a designation of ‘1’ for young fruit. Values are means of at least 3 replicates ± SD.

Semi-quantitative RT-PCR and RT-qPCR demonstrated that transcripts of all genes were detected in each tissue type, and changes in those patterns of expression were similar when recorded from either method (Figure 7; Figure S1). *GalLDH* and *GalDH* had similar patterns, being greatest in 40-DAA young fruits, and slightly diminished in mature leaves. Differences were not as evident within other tissues, although expression of *GalDH* was higher in the phloem than in young leaves or leaf and fruit petioles (Figure 7). Transcripts of *GPP*, *GMP*, *GalUR1*, *MDHAR*, *DHAR1*, and *DHAR2* were highest in mature leaves (source tissue); their levels clearly declined in 40-DAA fruits. In contrast, *GGP*, *GME*, and *GalUR3* expressions were greatest in young leaves. Although fruit petioles had higher AsA, expression of most detected genes was lowest there except for *GalUR3* and *DHAR1*. Other deviations from this pattern included higher expression of *GGP* in the leaf petioles, greater expression by *GME* and *GMP* in the phloem, and very low expression for *GalUR1*, *GalUR2*, and *DHARs* in young leaves (Figure 7).

Similarly, activities of GalLDH, GalDH, MDHAR, and DHAR were highest in the leaves, generally followed by young fruits; the exception was for MDHAR, which was more active in leaf petioles and young leaves than in immature fruits (Table S2). GalLDH and GalDH activities did not differ significantly among conducting-tissue types, and were the lowest in all tissues, whereas DHAR activity was higher in leaf and fruit petioles than in the phloem and young leaves (Table S2).

### Influence of ascorbate in fruit petioles on AsA content in fruits

Our experiments revealed that the fruit petiole had a high amount of AsA even though gene expression and enzyme activities were low in that tissue. Therefore, we examined whether ascorbate was being transported. Petioles from young (28-DAA) and mature (140-DAA) fruits were cultured *in vitro* in 10 mM AsA for 24 h. Afterward, no clear change in T-AsA content was found in either flesh or core, a main conducting tissue (Table 2). Meanwhile, continuously feeding the fruit petiole with 5 mM AsA, DHA, or L-GL for 15 d at the stages of young or ripening fruit led to a marked increase in AsA content in those petioles, but did not influence T-AsA levels in fruits at either stage (Table 3). Compared with fruits developing under normal (control) conditions, the petioles of 30-DAA fruits that had been half-girdled for 15 d showed damage to phloem transport from the leaves, but the T-AsA content was not altered (data not shown).

**Table 2:** T-AsA concentration (µmol·g^−1^·FW) in kiwi fruits after *in vitro* culture of fruit petioles in 10 mM AsA for 24 h.

	Young fruit		Mature fruit	
	Flesh	Core	Flesh	Core
Control	14.32±1.25a	4.32±0.33a	8.12±0.98a	3.52±0.32a
AsA	14.22±0.68a	4.46±0.42a	8.02±0.64a	3.46±0.28a

Values of T-AsA concentration are means of 5 replicates ± SD. Different letters within the same column indicate significant difference at P<0.05 by Duncan's test.

**Table 3:** T-AsA concentration (µmol·g^−1^·FW) in kiwi fruits after 5 mM AsA, DHA, or L-GL were added *in vivo* to fruit petioles at 3-d intervals for 15 d.

	Young fruit		Ripening fruit	
	Fruit petiole	Fruit	Fruit petiole	Fruit
Control	6.07±0.52c	13.38±0.85a	5.46±0.42b	7.89±0.52a
AsA	14.24±1.27a	13.99±0.83a	11.36±1.02a	8.17±0.83a
DHA	16.58±1.85a	13.63±1.12a	10.86±2.16a	7.92±0.94a
L-GL	7.86±0.83b	13.83±1.35a	5.58±0.96b	8.07±0.72a

Values of T-AsA content are means of 5 replicates ± SD. Different letters within the same column indicate significant difference at P<0.05 by Duncan's test.

However, ascorbate could be detected in phloem exudates collected from the petioles of source leaves or in 30-DAA fruit treated with 10 mM EDTA, with the AsA content being higher from the latter type. Compared with the control (treatment with 5 mM CaCl_2_ to induce callose gellation and reduce exudation), fruit petioles released less AsA whereas a dramatic decline in ascorbate was recorded from the leaf petioles (Table 4).

**Table 4:** T-AsA and AsA levels in fruit and leaf petiole exudates.

	Fruit petioles		Leaf petioles	
	10 mM EDTA	5 mM CaCl_2_	16 mM EDTA	5 mM CaCl_2_
T-AsA (nmol·cm−^1^ petiole)	68.7±5.27a	40.9±5.50b	25.9±5.61a	3.06±1.24b
AsA (nmol·cm−^1^ petiole)	52.6±5.56a	28.2±4.32b	15.9±3.07a	2.04±1.06b

Values are means of 5 replicates ± SD. For each value, different letters within the same tissue indicate significant difference at P<0.05 by Duncan's test.

### Histochemical localization of AsA in fruits

**Figure 8:**
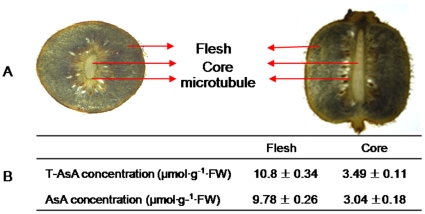
Ascorbate distribution within fruit. (A) histochemical localization using AgNO_3_, with AsA level indicated by black color reaction; (B) AsA contents in flesh and core. Values are means of 5 replicates ± SD.

To investigate AsA distribution within various tissues, we used the acidic-alcoholic AgNO_3_ method. A color reaction was positively proportional to AsA level. Signals were strongest in the flesh and weakest in the mesocarp. Microtubule cells had a much stronger color reaction, although the core was weaker than the flesh (Figure 8A). Finally, T-AsA and AsA contents were much higher in the flesh than in the core (Figure 8B).

**Figure 9:**
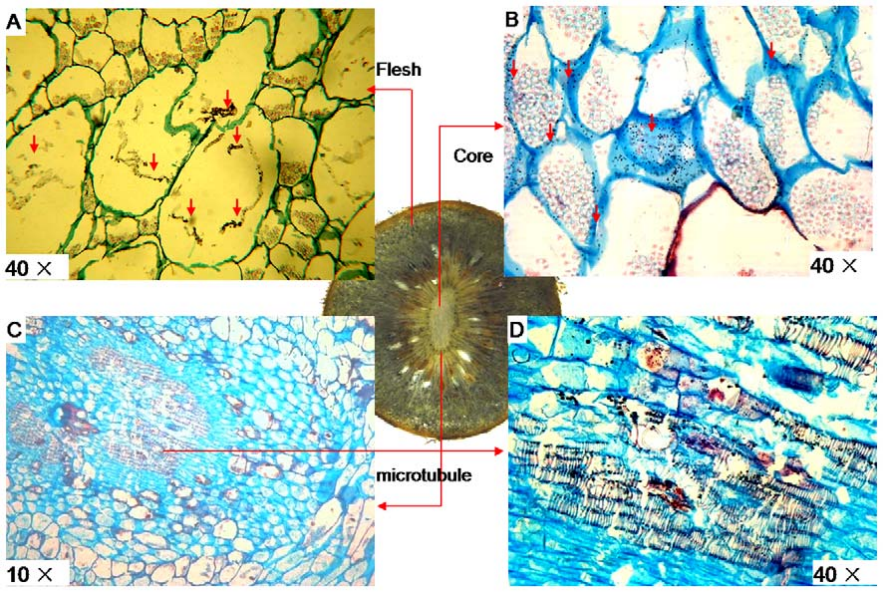
Histochemical localization of AsA per cool acidic-alcoholic AgNO_3_ method. Different cell types were sampled from fruit sections at 60 DAA. Dark spots indicate sites of deposition by metallic silver formed during AsA-dependent reduction of Ag^+^ ions. (A) 40× flesh cell; (B) 40× core cell; (C) 10× cell from microtubule zone; (D) 40× microtubule cell.

The flesh has two cell sizes; a dark deposit of metallic silver was clearly observed in the large cells but not in the small ones (a red deposit in those small cells could have been polyphenol). Small spots also were found on the walls of large cells (Figure 9). Numerous dark spots were visible on the walls of core cells, which had less AsA, but not on the interior of those cells (Figure 9B). Microtubules also revealed horseshoe-shaped spots, and many deposits of dark Ag^+^ ions were noted on their inner walls (Figure 9C, D). However, those dark deposits were not found on cells surrounding the microtubules.

## Discussion

### Formation and accumulation of ascorbate in kiwifruit

Bulley *et al.* [2] have reported that AsA contents in kiwifruit are highly regulated by developmental processes. Our results showed that ovaries after anthesis had less ascorbate and were more oxidized. This AsA pool increased over time, peaking at 30 DAA, and then decreased to Day 60 before remaining largely unchanged toward maturation. This suggests that, while ‘Qinmei’ fruits were developing in the first 30 d, the rate of AsA formation (based on fresh weight) exceeded the rate of oxidation loss (DHA degradation). However, as those fruits began to ripen, oxidation loss exceeded ascorbate production. This pattern is similar to that for *Actinidia chinensis* MP097 and MP212 [2]. Higher AsA contents also have been reported in the young fruits of acerola [24] and peach [35]. By comparison, such levels tend to increase over time in the fruits of tomato [3]. Therefore, regulation of AsA during developmental processes differs among species. Here, ascorbate steadily accumulated up to Day 45, and then remained fairly constant as the fruit matured. This has also been observed in the fruits of blackcurrant [25], where biosynthetic capacity is greater in the earlier stages.

When fruit discs representing different developmental stages were incubated with non-labeled putative substrates to synthesize ascorbate, D-Glc, L-Gal, and L-GL clearly enhanced AsA levels in 30-DAA discs, a response that has also been reported with pea seedlings [36]. This demonstrated that kiwi fruits are capable of AsA biosynthesis via the L-galactose pathway, and that the capacity to produce AsA is greater at Day 30 than at Day 75 or later, which is in agreement with patterns of AsA accumulation. L-GulL and D-GalUA also stimulated AsA levels in 30-DAA discs, indicating that young fruits utilize uronic acid (derivatives) for ascorbate synthesis [26], [36]. Although the enzyme that catalyzes L-GulL to AsA has not yet been identified in plant cells, Leferink *et al.* [37] have reported that recombinant GalLDH of *Arabidopsis thaliana* is not strictly specific to L-GL but can also oxidize L-GulL to AsA at significant rates. Therefore, future research should characterize kiwifruit GalLDH protein and assess its capacity for catalysis to L-GulL, based on our finding that AsA levels were less affected by feeding with L-GulL than with L-GL. Although the *Arabidopsis* genome contains several genes homologous to rat L-gulono-1,4-lactone oxidase (e.g., AT2G4676, AT5G11540), we found no apparent change in AsA content for T-DNA insertional mutations of AT2G4676 (our unpublished data).

Ascorbate is translocated from source to sink tissues in *Arabidopsis thaliana*, *Medicago sativa* [16], and *Solanum tuberosum* [17]. Here, the petioles from 40-DAA fruits had much more AsA than did the leaf petioles or phloem. However, except for *GalUR3*, those fruit petioles showed the lowest expression for genes encoding AsA-biosynthesis enzymes. The level of expression was in proportion to the amount of ascorbate that accumulated in each tissue type. Abundant AsA was measured in phloem exudates collected from the petioles of source leaves and 30-DAA fruits. It was also found in microtubule cells (Figure 9), after being located via the acidic-alcoholic AgNO3 method, which is specific to AsA at 4°C [34]. Therefore, our current results might lead us to conclude that long-distance transport of AsA occurs in *Actinidia*, and that its presence in the fruit could partly depend upon its movement from the leaves. However, both *in vitro* and *in vivo*, exogenous applications of AsA, DHA, or L-GL to the fruit petioles at young and mature stages did not enhance ascorbate in those fruits even though those contents were dramatically increased in the petioles. Because AsA deposits were not found in the cells surrounding the microtubules, it is possible that this region has an important role in unloading compounds from the microtubule tissues into the fruit flesh. These results suggest that AsA is not readily transported into the flesh and must, instead, be synthesized within that tissue, a process that has also been proposed with blackcurrant [25]. Differences in AsA distribution between large and small cells of the flesh also indicated that transport does not occur between the two types, and that the capacity for AsA biosynthesis is cell-specific in kiwifruit, despite the existence of 12 putative nucleobase-ascorbate transporters [38].

AsA localization to the vascular tissue has been observed histochemically in sections from potato stems and tubers [17] and apple fruits [26] that have been incubated with ethanolic AgNO_3_ under low temperature; those earlier results confirm the long-distance transport of AsA. However, the fact that cells around the vascular tissue have much less AsA than do the vascular cells, including those from potato stems [17] and apple fruit [26], implies that AsA can not unload well in tubers or fruits.

A series of AsA conjugates has been identified in the phloem of *Cucurbitaceae* members, with the most abundant being 6-O-glucosyl-L-ascorbic acid (6-GlcAsA) [39]. Based on all of these results, therefore, we conclude that the main role for AsA conjugates with glycosides in the phloem is the transport of those glycosides rather than of AsA. This conjugation with AsA (which has strong redox properties) increases glycoside stability. When carried into fruit, the conjugates are decomposed into glycoside and AsA before the former are unloaded into the flesh cells, so that AsA remains in the vascular tissue and fruit petioles as an antioxidant. This might explain why ascorbate is so abundant in kiwi fruit petioles and microtubule cells. In addition, AsA that has been localized with ethanolic AgNO_3_ in the vascular tissue might be partial AsA conjugates and not just AsA because those conjugates have redox properties similar to AsA [39]. Further study is necessary of the roles for these conjugates in plants.

### Regulation of AsA biosynthesis

As with the utilization of uronic acid (derivatives) by *Arabidopsis* for AsA synthesis [26], [36], D-GalUA could elevate ascorbate contents in young fruits and leaves of kiwi. Bulley *et al.* [2] have reported similar patterns of change in the expression levels of three *GalURs* that are clearly correlated with AsA content during kiwi fruit development. Nevertheless, we found that patterns varied among *GalUR1*, *GalUR2*, and *GalUR3* in different tissues, and they were not correlated with AsA levels. Apple roots have a very low AsA content but the highest expression of *GalUR* [40]. This shows that the degree of its expression is not an important factor in controlling ascorbate content, and that GalUR1, GalUR2, and GalUR3 have different functions among kiwifruit tissue types. Such findings conflict, however, with those reported for strawberry *GalUR*, a gene with the highest homology to kiwifruit *GalUR1* yet having expression that is highly correlated with AsA content [11].

In the most widely accepted L-galactose or Smirnoff–Wheeler pathway, GalLDH catalyzes the oxidation of the last precursor, L-GL, to AsA. It also could be required in a possible D-galacturonic acid pathway [23]. Its level of expression is well correlated with AsA contents in developing tissues and cells [15]. Here, the same was found for its association with AsA content during fruit development but not with the rate of accumulation. Different tissues also did not show a close relationship between expression and the amount of ascorbate. Some researchers have proposed that *GalLDH* is post-transcriptionally regulated and is not related to AsA content in different organs or under stress conditions [41], [42]. Silencing of this gene also does not lead to a clear decline in T-AsA contents in tomato leaves and fruits, although GalLDH activity is reduced by approximately 80% and cell enlargement is inhibited [43]. Therefore, these results suggest that expression of post-transcriptionally regulated *GalLDH* is not a main controlling factor of AsA levels, as has also been previously proposed by Linster and Clarke [1].

GalDH is a key enzyme that determines whether a plant can synthesize AsA via the L-galactose pathway [5]. Here, its transcripts and activity were correlated with ascorbate content in developed fruits, similar to that reported by Bulley *et al.* [2]. In 30-DAA fruits, GalDH expression was similar to that in mature leaves, perhaps because AsA was accumulating at that time. However, previous research has shown that ascorbate is substantially accumulated after L-galactose feeding [5], and that over-expression of *Arabidopsis GalDH* has no effect on AsA content in tobacco under natural conditions [8]. Hence, *GalDH* becomes a minor factor in controlling AsA synthesis via the L-galactose pathway.

In a pre-step of the L-galactose pathway, the level of *GME* transcripts was not highly correlated with AsA content and rates of accumulation during fruit development, c.f., 45 DAA versus 30 DAA. Nonetheless, phloem and young leaves, both tissues with the lowest amounts of ascorbate, had the greatest *GME* expression, suggesting that this gene has other functions besides AsA synthesis, such as non-cellulosic cell-wall biosynthesis, as reported in tomato [44]. Other studies also have shown that *GME* is not a control point for AsA synthesis in the fruits of kiwi [2] peach [35], tomato [3], or acerola [24]. *GMP* transcript levels showed a certain correlation with AsA content and accumulation rate during kiwi fruit development. GMP is required for the biogenesis of cell walls and protein glycosylation [23]. It is possible that those two processes also were associated with high expression in the phloem and mature leaves seen here.

During kiwi fruit development, the mRNA expression abundance of *GPP*, which catalyzes L-Gal-1-P to L-Gal [7], peaked in 15-DAA fruits, where AsA was most quickly accumulating. In contrast, expression levels for other genes were highest after 30 DAA, before clearly declining to 40 DAA. In different genotypes of kiwifruit, changes in GPP expression patterns show good correlation with rates of AsA accumulation, and *A. eriantha* (with a much higher AsA content) has a higher level of its expression than for GME and GGP [2]. Moreover, in two strains of *Chlorella pyrenoidosa* AsA hyper-accumulating mutants, activity appears to be up-regulated only for GPP, one of the L-galactose pathway enzymes [45]. Ioannidi *et al.* [3] also have found that GPP expression is closely associated with AsA content, and they propose that it plays an important role in regulating accumulation during the development and ripening of tomato fruits. We also have profiled the expression of genes involved in AsA biosynthesis in developing leaves [27] and fruits of apple (unpublished), and have observed, at the transcript level, a close relationship between GPP (in the L-galactose pathway) and ascorbate production. Additionally, its promoter extracted from young fruits of *A. deliciosa* has several different types of light-responsive motifs (our unpublished results). Therefore, future research should focus on analyzing the regulating characteristics of that promoter.

GPP partially purified from young fruits of *A. deliciosa* is very specific in its ability to hydrolyze L-gal-1-P [29]. However, Torabinejad *et* al. [46] reported that its protein of Arabidopsis was a bifunctional enzyme and can hydrolyze by L-gal-1-P and myo-inositol-1-phosphate. GPP has some homology with myo-inositol-1-phosphate phosphatase (MIPP), which catalyzes myo-inositol-1-phosphate to myo-inositol. Although IMPL-2, involved in histidine synthesis, is unable to catalyze L-Gal-1-P to L-Gal [47], IMPL-1 of *Arabidopsis* can hydrolyze by L-Gal-1-P to L-Gal, and can restore AsA biosynthesis when *GPP* is inhibited [47]. Therefore, this might explain why a GPP-knockout mutant of *Arabidopsis* is only partially deficient in AsA content [7]. Likewise, a greater amount of ascorbate that results from overexpression of purple acid phosphatase AtPAP15 [14] and myo-inositol oxidase [13] may cause a rise in MIPP and the hydrolysis of L-Gal-1-P. This is because myo-inositol cannot be used alone to produce AsA in plants [23]. Further study should investigate the functioning and characteristics of *GPP* in plant cells.

The gene for GGP (VTC2/GDP-L-Gal phosphorylase) also might play an important role in controlling AsA biosynthesis [1], [2]. Here, *GGP* expression was highest in 30-DAA fruits and then dramatically declined by 45 DAA, after which it remained at a constant level through fruit maturation, as reported by Bulley *et al.* [2]. Although this trend was similar to that for AsA content during kiwi fruit development, it did not parallel the rate of AsA accumulation. Ascorbate biosynthesis is the main reason for its accumulation in plant tissue [23], and its ultimate level is primarily determined by a balance between this synthesis and the oxidation loss of DHA. However, this accumulation only occurs within a certain phase of cellular development – in this case, during the young fruit stage, when the capacity is high for synthesis and low for recycling. Here, the rate of accumulation declined from 30 DAA while activities of MDHAR and DHAR, both of which recycle oxidized AsA, began to increase. Expression of GGP was highest at 30 DAA, suggesting that this gene does not control AsA synthesis at the transcript level. Our hypothesis was further supported by the greater expression of GGP in leaf petioles and young leaves, again showing that expression is irrelevant to AsA content. Although transformation methods are effective in elucidating the functions of genes, previous overexpression of GGP to enhance ascorbate content [2] did not demonstrate that it is indeed a regulatory gene for AsA biosynthesis. Overexpression of other pre-step genes in the L-galactose pathway, such as GDP-phosphomannose isomerase [48] and phosphomannomutase [49], might increase biosynthesis via a flux change in substrates when applied to transgenic plants. However, overexpression of other genes, e.g., GME, does not lead to altered amounts of ascorbate [2] because its expression alone in plant cells does not meet a minimum flux requirement for AsA biosynthesis.

MDHAR activity is correlated with AsA levels in tomato fruit under chilling stress [50]. It has also been implicated in the increment of DHA [51] as a fruit metabolite locus in tomato. Our results and those of others lend further support to these observations; *MDHAR* transcript and activity levels during fruit development were negatively correlated with AsA and DHA contents. Furthermore, in the later stages of maturation, transcripts and activity of MDHAR began to increase markedly while DHAR activity and its capacity for AsA biosynthesis showed a clear decline. MDHAR might play important roles in maintaining a higher AsA/DHA and in suppressing oxidation losses in kiwi fruits. In fact, that might be a main reason why such fruits exhibit high concentrations of ascorbate even after ripening. Because DHA cannot be reduced to AsA via DHAR, it is further oxidized to 2,3-diketogulonic acid or to non-renewable oxalic acid and/or tartaric acid before being lost [23]. Thus, DHAR is important for maintaining AsA levels and a redox state. Here, DHAR activity and the mRNA expression of *DHAR1* and *DHAR2* were greater in the fruit between Days 30 and 75, during which time biosynthetic capacity dramatically declined and MDHAR activity was lower. This demonstrates a complementary relationship between DHAR and MDHAR in maintaining a higher redox state of AsA. However, we found no clear association among AsA content, DHAR activity, and the mRNA expression levels of *DHAR1* and *DHAR2* during fruit development. Those genes also had different expression patterns at various stages of ripening and among tissue types, all of which might have been correlated with certain biological functions or enzyme localization.

### Conclusions

Ascorbate content in plants is highly regulated by developmental processes that may vary according to genotype, tissues, or cell type. Its biosynthesis is a main reason for its accumulation in many plant tissues, including fruits and conducting tissues. The extent of such accumulation is determined primarily by the balance between its synthesis and the oxidation loss of DHA. Our data indicated that levels of AsA in kiwi fruits are greatest at 30 DAA when it has higher biosynthetic capacity but lower expression and activities of MDHAR and DHAR, and then decrease up to 60 DAA, after which they decline only slightly. Expression of key genes involved in biosynthesis follow trends similar to that for AsA content, the exceptions being *GPP* and post-transcriptionally regulated *GalLDH*. However, GPP has good correlation with the rate of AsA accumulation whereas *GGP* does not. Expression of the latter is greater in leaf petioles and young leaves, both of which contain less ascorbate.

In conducting tissues, fruit petioles accumulate more AsA but expression is lowest for all but *GalUR3* and *DHAR1*. Using the ethanolic AgNO_3_ method, we found a large amount of AsA localized in the microtubules. However, it could not be transported from there into the fruits when exogenous AsA and DHA were added. Meanwhile, AsA distribution was cell-specific in the fruit flesh, with AsA being localized mainly in large cells. Therefore, we believe that *de novo* biosynthesis is the main reason for AsA accumulation in the fruits of cv. Qinmei because ascorbate is not easily transported and deposited into the fruit. In addition, GPP, but not GGP, is a good candidate for controlling AsA production. Studies of the promoters for these genes would bring new insight for the regulatory mechanisms of such biosynthesis in plants. Other future research should focus on degradation, including key factors that control ascorbate levels in apple plants. Of interest would be the extraction of AsA conjugates and functional analyses of the phloem and microtubules.

## Supporting Information

Figure S1 Differences in mRNA expression abundances of genes involved in AsA biosynthesis and recycling. Various tissue types were analyzed via semi-quantitative RT-PCR.
(0.11 MB TIF)

Table S1 Primers used in this study.
(0.05 MB DOC)

Table S2 Comparison of activities for GalLDH, GalDH, MDHAR, and DHAR among different tissues of kiwifruit sampled at 30 DAA.
(0.03 MB DOC)
