# Supplementary figures and images for: Ascorbate Biosynthesis during Early Fruit Development Is the Main Reason for Its Accumulation in Kiwi
Source: PLoS One. 2010 Dec 9;5(12):e14281. doi: 10.1371/journal.pone.0014281 (PMC3000333; doi:10.1371/journal.pone.0014281)

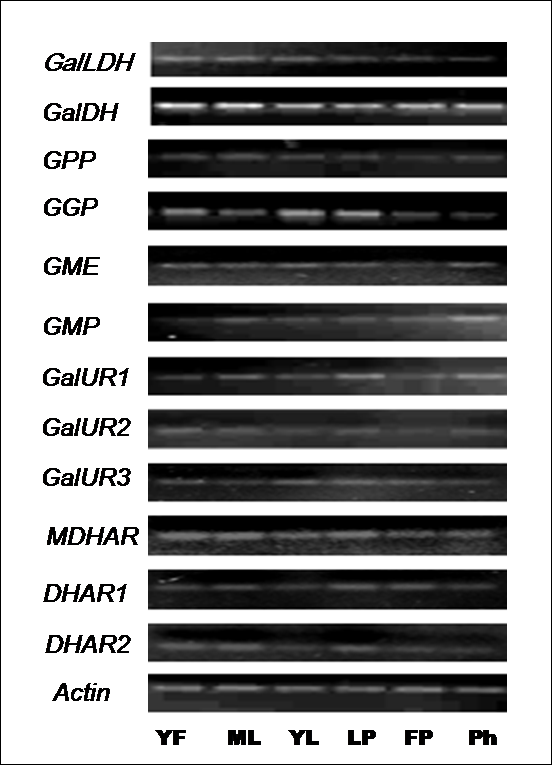

Supplement: Figure S1 — Differences in mRNA expression abundances of genes involved in AsA biosynthesis and recycling. Various tissue types were analyzed via semi-quantitative RT-PCR. (0.11 MB TIF) [file pone.0014281.s001.tif]
