# Supplementary material for: Ascorbate Biosynthesis during Early Fruit Development Is the Main Reason for Its Accumulation in Kiwi
Source: PLoS One. 2010 Dec 9;5(12):e14281. doi: 10.1371/journal.pone.0014281 (PMC3000333; doi:10.1371/journal.pone.0014281)
Supplement: Table S1 — Primers used in this study. (0.05 MB DOC) [file pone.0014281.s002.doc]

**Table S1.** Oligonucleotide sequences for primers used in this study (f and r, forward and reverse).

| **Gene** | **Primer** | **Sequence (5’-3’)** | **Accession No.** |
| --- | --- | --- | --- |
| *GalLDH* | GalLDHf | CGCCAGAGGAAAGAGATAACAGA | GU339039 |
|  | GalLDHr | CCCAATGTTCGTAAGCAGAAAAC |  |
| *GalDH* | GalDHf | TTGGGAAGGATGCGAAAAC | EU525846 |
|  | GalDHr | GCAGCCACTTCACAAAGCA |  |
| *GPP* | GPPf | GCCTCTTGATGGGACAACTAACT | AY787585 |
|  | GPPr | CGTTCAGACTGGGATGACAACTT |  |
| *GGP* | GGPf | CGGCAACAACATACCTTACAAT | GU339036 |
|  | GGPr | CTTCAGACGCCTCCTCATAGT |  |
| *GME* | GMEf | CGGATTGAATGTAGGATTGGAA | GU339037 |
|  | GMEr | CATCAGTGGAGGTAAGGGTCT |  |
| *GMP* | GMPf | TATTGGAGTAAGCGAAGTGGTT | FJ643600 |
|  | GMPr | TGTCGATGAGTTTGTCCCTAG |  |
| *GalUR1* | GalUR1f | GATGCCGGACCTGGAAACT | GU339035 |
|  | GalUR1r | ACTTGATGATGCCGAGATGG |  |
| *GalUR2* | GalUR2f | AGGTGGCTCTGAGGTGGGTGTAT | GU339038 |
|  | GalUR2r | CCAAAATAGAAGCTAAGGTAACGCC |  |
| *GalUR3* | GalUR3f | GTGAAACCAGGGGCTTACGAATAC | FG4889261 |
|  | GalUR3r | ATTGACAGCAGGAGGGATGGTTG |  |
| *MDHAR* | MDHARf | TCAAATTATCAAGGGAACTGTGGC | GU339041 |
|  | MDHARr | ACCAACAACAACGATGTCCGCT |  |
| *DHAR1* | DHAR1f | GCCGCTTCTGGTTCTCCTGAT | GU339034 |
|  | DHAR1r | TCGGAGTCAGGAATCCATTTGTC |  |
| *DHAR2* | DHAR2f | GCGACTATCAAACACCTCACCG | GU339040 |
|  | DHAR2r | ATAGCTTGTTGGGGACCGTGAC |  |
| *Actin* | Actinf | GCTTACAGAGGCACCACTCAACC | EF063572 |
|  | Actinr | CCGGAATCCAGCACAATACCAG |  |
| *18S RNA* | 18sf | GTCGTAACAAGGTTTCCGTAGGT | AB253775 |
|  | 18sr | CAAAGGGAAGAAAGAGTAGGGTT |  |
