# Supplementary material for: Ascorbate Biosynthesis during Early Fruit Development Is the Main Reason for Its Accumulation in Kiwi
Source: PLoS One. 2010 Dec 9;5(12):e14281. doi: 10.1371/journal.pone.0014281 (PMC3000333; doi:10.1371/journal.pone.0014281)
Supplement: Table S2 — Comparison of activities for GalLDH, GalDH, MDHAR, and DHAR among different tissues of kiwifruit sampled at 30 DAA. (0.03 MB DOC) [file pone.0014281.s003.doc]

**Table S2.** Comparison of activities for GalLDH, GalDH, MDHAR, and DHAR among different tissues of kiwifruit sampled at 30 DAA.

|  | **GalLDH** | **GalDH** | **MDHAR** | **DHAR** |
| --- | --- | --- | --- | --- |
| Young fruit | 4.25 ± 0.21b | 8.76 ± 0.48b | 4.54 ± 0.32c | 13.63 ± 0.76b |
| Mature leaf | 5.32± 0.37a | 12.12 ± 1.22a | 9.63 ± 1.23a | 17.22 ± 2.22a |
| Young leaf | 3.02 ± 0.21c | 4.85 ± 0.18c | 5.23 ± 0.29b | 7.36 ± 1.05d |
| Leaf petiole | 1.46 ± 0.12d | 2.14 ± 0.18d | 5.45 ± 0.48b | 10.95 ± 0.54c |
| Fruit petiole | 1.18 ± 0.34d | 1.42 ± 0.21e | 4.26 ± 0.32c | 11.45 ± 0.84c |
| Phloem | 1.35 ± 0.16d | 1.98 ± 0.19d | 4.04 ± 0.35c | 7.54 ± 0.75d |

Values of AsA levels are means of 5 replicates ± SD. Different letters within the same column indicate significant difference at P < 0.05 by Duncan’s test.
